# HIF1α/HIF2α induces glioma cell dedifferentiation into cancer stem cells through Sox2 under hypoxic conditions

**DOI:** 10.7150/jca.54402

**Published:** 2022-01-01

**Authors:** Pan Wang, Sheng Gong, Bin Liao, Jinyu Pan, Junwei Wang, Dewei Zou, Lu Zhao, Shuanglong Xiong, Yangmin Deng, Qian Yan, Nan Wu

**Affiliations:** 1 Chongqing Medical University, Chongqing 400016, China; 2 Department of Neurosurgery, Chongqing General Hospital, University of Chinese Academy of Sciences, Chongqing 401147, China; 3 Department of Oncology, Chongqing University Cancer Hospital, Chongqing 400030, China *Correspondence: Dr. Nan Wu, mailing address: No. 1 Yixueyuan Road, Yuzhong District, Chongqing, 400016, P. R. China. Tel. and Fax: +86 23 63512096. E-mail: wunan881@tmmu.edu.cn

**Keywords:** glioma stem cells, dedifferentiation, hypoxia, HIF1α/HIF2α, Sox2

## Abstract

**Objective:** Our previous study showed that glioma stem-like cells could be induced to undergo dedifferentiation under hypoxic conditions, but the mechanism requires further study. HIF1α and HIF2α are the main molecules involved in the response to hypoxia, and Sox2, as a retroelement, plays an important role in the formation of induced pluripotent stem cells, especially in hypoxic microenvironments. Therefore, we performed a series of experiments to verify whether HIF1α, HIF2α and Sox2 regulated glioma cell dedifferentiation under hypoxic conditions.

**Materials and methods:** Sphere formation by single glioma cells was observed, and CD133 and CD15 expression was compared between the normoxic and hypoxic groups. HIF1α, HIF2α, and Sox2 expression was detected using the CGGA database, and the correlation among HIF1α, HIF2α and Sox2 levels was analyzed. We knocked out HIF1α, HIF2α and Sox2 in glioma cells and cultured them under hypoxic conditions to detect CD133 and CD15 expression. The above cells were implanted into mouse brains to analyze tumor volume and survival time.

**Results:** New spheres were formed from single glioma cells in 1% O_2_, but no spheres were formed in 21% O_2_. The cells cultured in 1% O_2_ highly expressed CD133 and CD15 and had a lower apoptosis rate. The CGGA database showed HIF1α and HIF2α expression in glioma. Knocking out HIF1α or HIF2α led to a decrease in CD133 and CD15 expression and inhibited sphere formation under hypoxic conditions. Moreover, tumor volume and weight decreased after HIF1α or HIF2α knockout with the same temozolomide treatment. Sox2 was also highly expressed in glioma, and there was a positive correlation between the HIF1α/HIF2α and Sox2 expression levels. Sox2 was expressed at lower levels after HIF1α or HIF2α was knocked out. Then, Sox2 was knocked out, and we found that CD133 and CD15 expression was decreased. Moreover, a lower sphere formation rate, higher apoptosis rate, lower tumor formation rate and longer survival time after temozolomide treatment were detected in the Sox2 knockout cells.

**Conclusion:** In a hypoxic microenvironment, the HIF1α/HIF2α-Sox2 network induced the formation of glioma stem cells through the dedifferentiation of differentiated glioma cells, thus promoting glioma cell chemoresistance. This study demonstrates that both HIF1α and HIF2α, as genes upstream of Sox2, regulate the malignant progression of glioma through dedifferentiation.

## Introduction

Glioma stem cells (GSCs) were identified many years ago^1^, and studies have suggested that GSCs play an important role in malignant glioma progression^2^, ^3^. Many factors, such as hypoxia, contribute to GSC stemness maintenance, thus promoting glioma chemoresistance and malignant progression^4^-^6^. In addition, hypoxia functions as an inducer that may promote differentiated glioma cell dedifferentiation and induce GSC formation. For example, in 2007, Blazek^7^ and Platet^8^ cultured unsorted glioma cells under hypoxic conditions and found an increased proportion of CD133^+^ cells. In their opinion, two reasons led to this: one was that GSCs proliferated faster under hypoxic conditions than differentiated cancer cells, and the other was the dedifferentiation process that differentiated glioma cells undergo in a hypoxic environment. We agree with the opinion of Bar^4^, who thought hypoxia influenced the ratio of symmetrical and asymmetrical division of GSCs, thus altering the frequency of stem-like properties in the daughter cells and leading to an increase in the GSC ratio in newly formed tumors. However, we still cannot rule out the possibility of GSCs undergoing a dedifferentiation process under hypoxic conditions from differentiated glioma cells. To address this query, we performed a series of experiments and found that GSCs with relatively high expression of stem cell markers, such as CD133 and CD15, could be induced to undergo dedifferentiation in a hypoxic microenvironment from differentiated glioma cells. Studies have demonstrated that the molecules involved in the response to hypoxia in glioma are mainly hypoxia-inducible factor-1α (HIF1α) and hypoxia-inducible factor-2α (HIF2α), which lead to chemoresistance by maintaining GSC stemness^9^-^11^. However, little is known about whether HIF1α and HIF2α regulate dedifferentiation, and we detected the detailed mechanism, which is reported in this article.

Induced pluripotent stem cells (iPSCs) have been successfully generated through reprogramming Sox-2, Oct-4, Klf-4, Nanog, lin28A and lin28B in cells, such as human dermal fibroblasts^12^, ^13^, especially under hypoxic conditions^14^, ^15^. In addition, recent studies have suggested that recombination of Sox-2, Oct-4, Klf-4, Nanog, lin28A and lin28B in tumor cells, such as lung adenocarcinoma cancer^16^, melanoma^17^ and pancreatic cancer cells^18^, also induces stemness features with relatively high expression of stem cell markers and a relatively strong tumorigenesis capacity. Therefore, we wondered whether these factors could induce the formation of GSCs and found that Sox2 was highly expressed in glioma according to The Cancer Genome Atlas (TCGA) database and promoted the dedifferentiation process in a hypoxic environment. Regarding the regulatory relationship among HIF1α, HIF2α and Sox2, studies have suggested that HIF1α plays an important role in upregulating Sox2 expression 19. However, there are few studies on the relationship between HIF2α and Sox2 in glioma, let alone whether these two factors can induce glioma cell dedifferentiation under hypoxic conditions. We addressed this question and performed a series of studies to verify that both HIF1α and HIF2α regulate glioma cell dedifferentiation in a hypoxic microenvironment through Sox2.

## Materials and Methods

### Public data collection

The Cancer Genome Atlas (TCGA), Genotype-Tissue Expression (GTEx), Cancer Cell Line Encyclopedia (CCLE) and Chinese Glioma Genome Atlas (CGGA) databases can be found at http://cancergenome.nih.gov/, https://www.gtexportal.org/, https://portals.broadinstitute.org/ccle, and http://www.cgga.org.cn, respectively. The TCGA and CGGA databases were used to analyze the expression of HIF1α, HIF2α, Sox2, Oct4, KLF4, Nanog, Lin28A and Lin28B through Gene Expression Profiling Interactive Analysis (GEPIA) (http://gepia.cancer-pku.cn/detail.php) and CGGA (http://www.cgga.org.cn/analyse/RNA-data.jsp). The TCGA and GTEx databases were used to analyze the correlations among HIF1α, HIF2α and Sox2 through the dplyr, tibble and ggpolt2 packages in R.

### Cell isolation, cell culture and cell sorting

GL261 cells were obtained from ATCC, and primary glioma cells were isolated from surgical waste. The primary glioma tissue samples were initially minced and digested with 0.25% trypsin (HyClone, USA) and 10 U/ml DNase I (Sigma, USA) at 37°C for 45~60 min. ACK lysis buffer (Beyotime Biotechnology, China) was used to lyse red blood cells. The suspension was washed with PBS two times and filtered through a 100-μm cell strainer. The obtained cells were cultivated in DMEM/F12 (HyClone, USA) supplemented with 10% fetal bovine serum (FBS; Gibco, USA) to maintain growth in 21% O_2_. The surgical waste obtained from patients was anonymized. After culturing these cells for approximately 3~5 days, we sorted CD133^-^CD15^-^ cells by magnetic activated cell sorting (MACS). In brief, cell suspensions were prepared after 0.25% trypsin digestion by resuspension in PBS containing 0.08% EDTA and 0.5% BSA (PBSE; 10^8^ cells/500 μl) and then incubated at 4°C for 15 min with polyclonal rabbit anti-human/mouse CD133^+^ IgGs (Miltenyi Biotech, Germany). The suspension was washed with PBS containing 1% BSA, resuspended in PBSE (10^8^ cells/300 μl), labeled with goat anti-rabbit IgG microbeads (Miltenyi Biotech, Germany), cultured at 10°C for another 15 min, washed and resuspended in 500 μl of PBSE. A cell separation column with a flow resistor was placed in a miniMACS magnet that was fixed on a MACS multistand and flushed with 500 μl of PBSE. The cell suspension was poured into the column reservoir, and unlabeled, nonmagnetic CD133^-^ cells that passed through the column were collected. The above steps were repeated three times to increase the purity of the collected CD133^-^ cells. Identical steps were used to sort CD15^-^ cells from CD133^-^ cells. Sorted CD133^-^CD15^-^ cells were cultured in DMEM/F12 supplemented with 10% FBS (DMEM/F12+10% FBS) to maintain their differentiation status.

### Clonogenicity of single glioma cells under hypoxic conditions

Suspensions were made from glioblastoma multiforme (GBM) and GL261 cells in DMEM/F12+10% FBS with a density of 1,500 cells/1 ml medium. One microliter of cell suspension was placed into each well of 96-well plates that contained 170 μl of serum-free DMEM/F12. Six 96-well plates were utilized for each cell line, and two groups for each cell line were randomly grouped with three 96-well plates. One group was incubated in 1% O_2,_ and the other was incubated in 21% O_2_. Sphere formation was observed, imaged and recorded on days 0, 3, 7, 14 and 21.

### Immunofluorescence detection

New spheres formed by single glioma cells, GBM cells or GL261 cells cultured in 1% O_2_ for 72 h were evaluated for HIF1α, HIF2α and Sox2 expression using immunofluorescence. Cells were fixed with 4% paraformaldehyde at 4°C for 30 min, washed with PBS containing 0.5% Triton X-100 (Sigma, USA), and blocked with 10% serum. The cells were incubated at 4°C for 24 h with primary antibodies against HIF1α (1:100, R&D Systems, USA), HIF2α (1:100, R&D Systems, USA), SOX-2 (1:100, R&D Systems, USA), CD133 (1:150, MyBiosource, USA) and CD15 (1:100, R&D Systems, USA). The cells were then washed with PBS three times and incubated for 2 h with fluorophore-labeled secondary antibodies (CST, USA), and images were acquired using a laser scanning confocal microscope (LSM780, ZEISS, Germany).

### Western blotting detection

New spheres formed by single glioma cells and CD133^-^CD15^-^ cells cultured in 21% O_2_ (control group) or 1% O_2_ for 72 h were evaluated for HIF1α, HIF2α, SOX-2, CD133 and CD15 expression by western blotting. Total protein was prepared using prechilled RIPA buffer (Beyotime Biotechnology, China), and the isolated proteins were subjected to SDS-PAGE, followed by transfer to nitrocellulose membranes. Then, the membranes were blocked with 5% nonfat milk and incubated with primary antibodies against HIF1α (1:1,000, R&D Systems, USA), HIF2α (1:1,000, R&D Systems, USA), SOX-2 (1:1,000, R&D Systems, USA), CD133 (1:1,000, MyBiosource, USA) and CD15 (1:1,000, R&D Systems, USA) at 4°C overnight. HRP-labeled secondary antibodies (Beyotime Biotechnology, China) were added to the membranes and incubated at 37°C for 1 h. Enhanced chemiluminescence was utilized for visualization.

### Real-time quantitative polymerase chain reaction (RT-qPCR)

New spheres formed by single glioma cells and control cells in 21% O_2_ for 12 h were used to detect RNA expression by RT-qPCR. Total RNA was prepared using TRIzol (Invitrogen, USA) according to the manufacturer's instructions. The melting conditions were 94°C for 5 min, the denaturing conditions were 94°C for 30 s, the annealing conditions were 57°C for 30 s, and the extension conditions were 72°C for 30 s. These conditions were used for a total of 40 cycles. The primer sequences are presented in Supplementary Table S1.

### Flow cytometry (FCM) analysis

Cells were exposed to 21% O_2_ or 1% O_2_ for 48 h, and temozolomide (TMZ) (400 μM) was added to the culture medium for an additional 72 h before cell apoptosis was detected using FCM. Cells were digested with 0.25% trypsin and prepared as a single-cell suspension in PBS at a density of 1×10^6^ cells/ml, and 100 μl of suspension was added to an Eppendorf tube. The cells were centrifuged, and 195 μl of 0.05% trisodium citrate-dihydrate and 5 μl of Annexin V-FITC were added to the cell suspension and incubated in the dark for 15 min. Then, 190 μl of 0.05% trisodium citrate-dihydrate and 10 μl of propidium iodide (PI) were added to the centrifuged cell suspension and incubated for 10 min in the dark. Samples were analyzed using a flow cytometer (BD Accuri C6, Germany).

### In vivo study

Primary glioma cells (8×10^4^) were seeded into mouse brains, and the mice were housed in 10% O_2_ for 14 days. Tumor and normal tissues were collected to analyze the expression of HIF1α, HIF2α, Sox2, CD133 and CD15 using immunohistochemistry (IHC). The detailed steps for IHC are described below.

### Immunohistochemical detection

HIF1α, HIF2α, Sox2, CD133 and CD15 expression in tumor or normal tissues obtained from mouse brains was detected by IHC. Briefly, tissue samples were fixed in formalin and embedded in paraffin, followed by dewaxing in xylene, rinsing in a graded ethanol series and rehydration in double-distilled water. The slides were washed with PBS for 3 min, immunostained using primary antibodies against HIF1α (1:100, R&D Systems, USA), HIF2α (1:100, R&D Systems, USA), SOX-2 (1:100, R&D Systems, USA), CD133 (1:150, MyBiosource, USA) and CD15 (1:100, R&D Systems, USA), and incubated at 4°C overnight. The slides were washed with PBS buffer again and covered with an HRP-labeled polymer for 2 h. Then, the tissue samples were covered with a DAB chromogen solution and incubated for ~1 min to allow staining reactions to occur, and images were acquired.

### HIF and Sox2 knockout assays

HIF1α, HIF2α and Sox2 sgRNAs for plasmid constructs were designed based on the CRISPR design program (http://crispr.mit.edu), annealed and cloned into the lentiCRISPRv2 vector (Addgene, #52961, USA); the sgRNA oligonucleotide sequences are listed in Supplementary Table S2. The constructed lentiviruses were transfected into 293T cells with a transducing vector and the packaging vectors psPAX2 (Addgene #12260, USA) and pMD2. G (Addgene #12259, USA). After 48 h of transfection, the supernatant containing viral particles was collected, filtered and transduced into GL261 and GBM cells. Western blotting was used to confirm the knockout of HIFs and Sox2. To determine the influence of low HIF1α, HIF2α and Sox2 expression on glioma cells, GL261 and GBM cells were cultured in the presence of TMZ in 1% O_2_ before apoptosis was detected. The detailed steps were described earlier in the manuscript.

Next, the cells described above (8×10^4^) were injected into the brains of mice, and the mice were housed in 10% O_2_. Next, these mice were randomly grouped (Con+TMZ, Vector+TMZ, HIF1α-ko+TMZ, HIF2α-ko+TMZ, HIF1α/HIF2α-ko+TMZ and Sox2-ko+TMZ). TMZ (2 mg/kg) was injected into the enterocoelia each day from day 3 to day 17. Magnetic resonance imaging (MRI) was used to measure tumor volume on day 21 for five mice, and the other mice were used to record survival time.

### Statistical analysis

Data are presented as the mean±standard deviation (SD), and we used SPSS 19.0 for statistical analysis. The significance of differences between two groups was determined using Student's t test, and one-way analysis of variance (one-way ANOVA) was utilized for comparing at least three groups. The log-rank test was performed for survival analysis. Pearson's correlation was used to analyze the correlations among HIF1α, HIF2α and Sox2. *P*<0.05 was considered to be statistically significant.

## Results

### Hypoxia induced the dedifferentiation of differentiated glioma cells into GSCs

**Figure 1:**
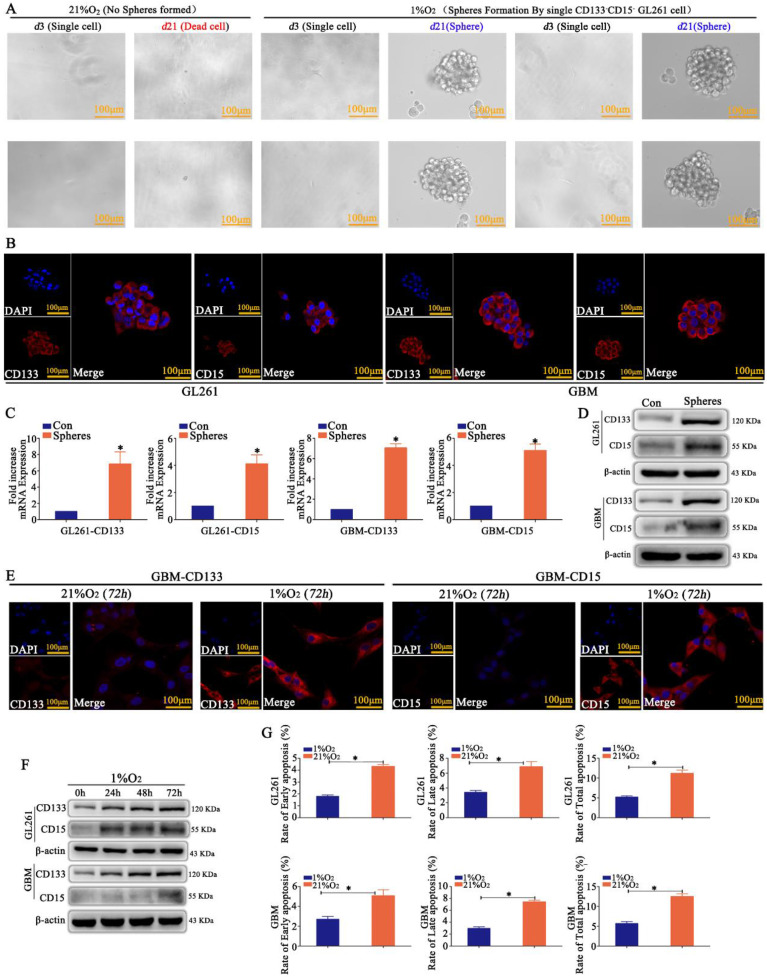
** Hypoxia induced the dedifferentiation of glioma cells A** A single CD133^-^CD15^-^ GL261 cell line was plated in each well of 96-well plates, and the cells were cultured in 21% O_2_ or 1% O_2_ for 21 days. The results revealed that the cells died in 21% O_2_; however, the survived cells in 1% O_2_ formed spheres in suspension.** B-D** The newly formed spheres highly expressed CD133 and CD15, as determined by immunofluorescence, RT-qPCR and western blotting; however, there was no expression in the control group cultured in 21% O_2_ for 72 h. **E** CD133^-^CD15^-^ primary glioma cells presented high expression of CD133 and CD15 after culturing in 1% O_2_ for 72 h, but there was no expression in the cells cultured in 21% O_2_ for 72 h. **F** CD133 and CD15 expression increased significantly in a time-dependent manner in CD133^-^CD15^-^ primary and GL261 cells cultured in 1% O_2_. **G** Cell apoptosis detection showed that there were higher apoptosis rates in cells cultured in 21% O_2_ than in cells cultured in 1% O_2_ after TMZ treatment (^*^P<0.05, independent-samples t test).

CD133^-^CD15^-^ GL261 and primary glioma cells were sorted by MACS, and just one cell was plated in each well of 96-well plates and incubated in 21% O_2_ or 1% O_2_ for 21 days. We found that most of the cells died in 21% O_2_; however, the cells in 1% O_2_ appeared to be in a state of suspended spheres, and the sphere formation rate was over 95% after hypoxic exposure for 21 days according to the statistical analysis (Fig 1A and Supplementary Figure S1A). Immunofluorescence detection showed that these newly formed spheres highly expressed CD133 and CD15 (Fig 1B). RT-qPCR and western blotting demonstrated the same results for spheres; however, the control cells cultured in 21% O_2_ for 72 h did not express CD133 or CD15 (Fig 1C-D). CD133^-^CD15^-^ GL261 and primary glioma cells were then cultured in 1% O_2_ for 72 h, and the results revealed that these cells highly expressed CD133 and CD15 (Fig 1E and Supplementary Figure S1B), and cells cultured in 1% O_2_ for 24, 48 or 72 h highly expressed CD133 and CD15 in a time-dependent manner under hypoxic conditions (Fig 1F). Finally, CD133^-^CD15^-^ GL261 and primary glioma cells were cultured in 21% O_2_ or 1% O_2_ for 48 h, TMZ (400 μM) was added to the culture medium for an additional 72 h, and cell apoptosis was detected using FCM. The results showed that there were higher early, late and total apoptosis rates for the cells cultured in 21% O_2_ than for those cultured in 1% O_2_ (Fig 1G).

### HIF1α/HIF2α were highly expressed in glioma

**Figure 2:**
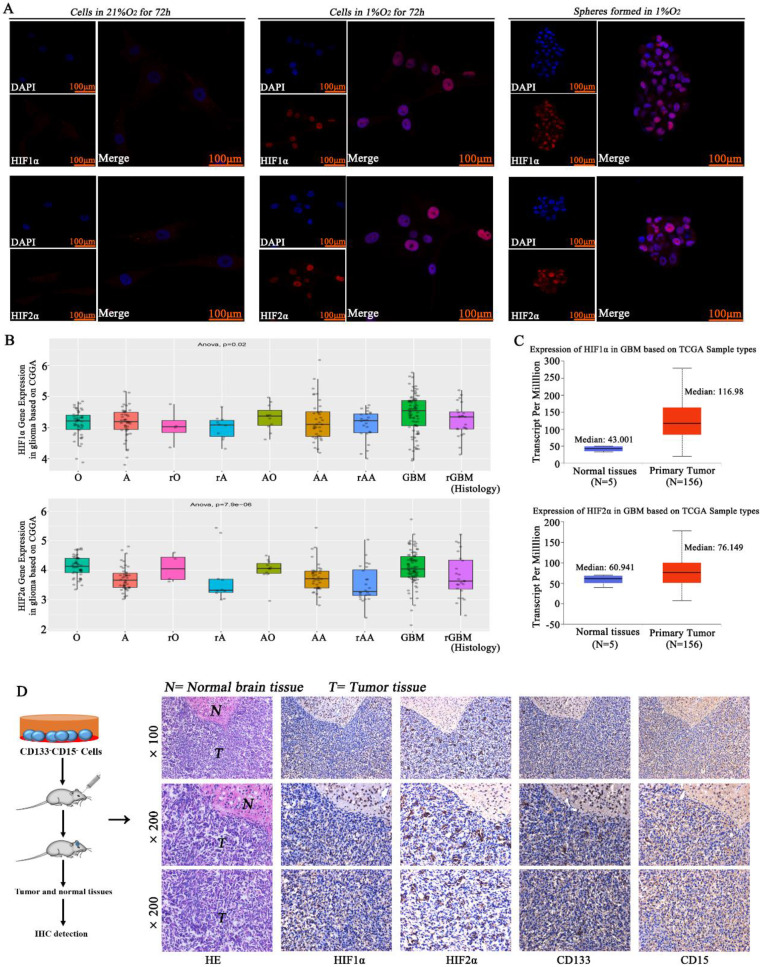
** HIF1α/HIF2α were highly expressed in glioma A** CD133^-^CD15^-^ primary glioma cells were cultured in 21% O_2_ or 1% O_2_, and there was no HIF1α or HIF2α expression with normoxic culture, but the cells cultured in 1% O_2_ had much higher expression of HIF1α and HIF2α. The spheres formed in 1% O_2_ also highly expressed HIF1α and HIF2α. **B** The CGGA database showed that glioma tissues highly expressed HIF1α and HIF2α. **C** The TCGA database showed that HIF1α and HIF2α were highly expressed in glioma tissues.** D** CD133^-^CD15^-^ primary glioma cells were seeded into the brains of mice, and the mice were housed in a normoxic environment. Glioma tissues were collected, and the expression of HIF1α, HIF2α, CD133 and CD15 was detected. The results showed that all the proteins above were highly expressed in glioma tissues (^*^P<0.05, ^#^*P*>0.05, independent-samples t test).

CD133^-^CD15^-^ GL261 and primary glioma cells were cultured in 21% O_2_ or 1% O_2_, and the results showed that there was no HIF1α or HIF2α expression in the normoxic environment, but the cells in 1% O_2_ had much higher expression of HIF1α and HIF2α. The spheres formed in 1% O_2_ by single CD133^-^CD15^-^ cells also highly expressed HIF1α and HIF2α (Fig 2A). By using the CGGA database, we found that almost all kinds of glioma pathologies highly expressed HIF1α and HIF2α (Fig 2B and supplementary table S3). By using the TCGA and GTEx databases, we found that both HIF1α and HIF2α were highly expressed in glioma tissues (Fig 2C). We then implanted CD133^-^CD15^-^ primary glioma cells into mouse brains and housed the mice for 14 days. We collected tumor tissues, and the results demonstrated that HIF1α, HIF2α, CD133 and CD15 were highly expressed in these glioma tissues (Fig 2D).

### HIF1α/HIF2α regulated glioma cell dedifferentiation under hypoxic conditions

**Figure 3:**
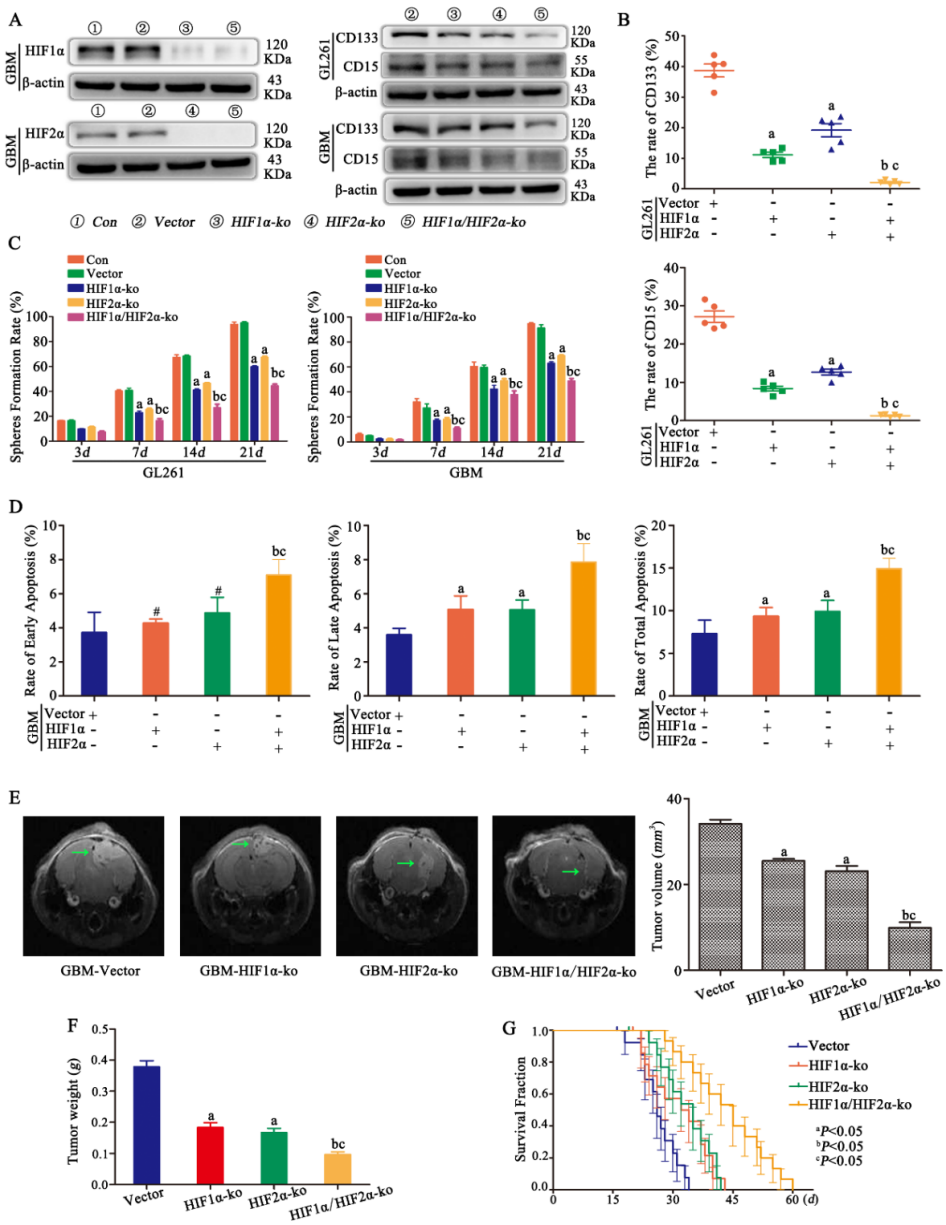
** HIF1α/HIF2α regulated glioma cell dedifferentiation under hypoxic conditions A** CD133^-^CD15^-^ primary glioma cells with HIF1α and/or HIF2α knocked out were successfully established. The results showed that CD133 and CD15 expression were decreased significantly after HIF1α or HIF2α knockout, and cells with both HIF1α and HIF2α knocked out presented the lowest expression of CD133 and CD15. **B** FCM detection showed that the proportions of CD133^+^ and CD15^+^ cells among HIF1α-ko or HIF2α-ko cells were lower than those among vector cells cultured in 1% O_2_ for 7 days. Moreover, the CD133^-^ and CD15^-^positive expression rates of the group containing cells with HIF1α and HIF2α simultaneously knockout were much lower than those of the other three groups (the vector, HIF1α-ko and HIF2α-ko groups). **C** The sphere formation rates of HIF1α-ko and HIF2α-ko group cells were lower than those of vector group cells, and after knocking out both HIF1α and HIF2α simultaneously, the sphere formation rate reached the lowest level. **D** HIF1α-ko cells or HIF2α-ko cells in 1% O_2_ had higher late and total apoptosis rates than control cells. In addition, after simultaneous HIF1α and HIF2α knockout, the rates of early, late and total apoptosis were significantly higher than those following the other transfections. **E-F** Vector, HIF1α-ko, HIF2α-ko or HIF1α/HIF2α-ko cells (8×10^4^) were implanted into the mouse brain, and tumor volume and weight were reduced with HIF1α or HIF2α knockout under the same TMZ treatment. The group with both HIF1α and HIF2α knocked out presented the smallest tumor volume **G** The group with HIF1α-ko, HIF2α-ko had longer survival time and the group with HIF1α-ko and HIF2α-ko simultaneously had the longest survival time compared with other three groups. (^a^*P*<0.05 represents differences between the vector control group and the HIF1α- or HIF2α-knockout alone groups. ^b^*P*<0.05 represents the difference between the vector control group and HIF1α/HIF2α-knockout group. ^c^*P*<0.05 represents differences between the HIF1α/HIF2α-knockout group and the HIF1α- or HIF2α-knockout alone group. ^#^*P*>0.05 represents differences between the vector control group and HIF1α- or HIF2α-knockout alone groups. One-way ANOVA).

HIF1α and HIF2α were successfully knocked out in CD133^-^CD15^-^ primary glioma cells, and the same result was found for CD133^-^CD15^-^ GL261 cells (data not shown). Vector, HIF1α-knockout (ko), HIF2α-ko and HIF1α/HIF2α-ko cells were cultured in 1% O_2_ for 72 h, and the results showed that CD133 and CD15 expression decreased significantly after HIF1α or HIF2α knockout and that both HIF1α and HIF2α knockout cells presented the lowest expression of CD133 and CD15 (Fig 3A). FCM was used to detect the proportion of CD133^+^CD15^+^ cells after culturing CD133^-^CD15^-^ glioma cells in 1% O_2_ for 7 days, and the results showed that both CD133 expression and CD15 expression were significantly decreased in HIF1α-ko and HIF2α-ko cells compared with vector cells. The CD133- and CD15-positive expression rates of the HIF1α/HIF2α-ko group cells were much lower than those of the other three groups (Fig 3B). This result meant that knocking out HIF1α and HIF2α simultaneously produced the strongest capacity to block CD133 and CD15 expression. Next, sphere formation by single CD133^-^CD15^-^ primary or GL261 glioma cells after HIF1α or HIF2α knockout was detected. According to the results, the sphere formation rates of HIF1α-ko or HIF2α-ko group cells were decreased compared with those of vector group cells, and after both HIF1α and HIF2α were knocked out at the same time, the sphere formation rate reached the lowest level (Fig 3C). Next, we cultured vector, HIF1α-ko, HIF2α-ko and HIF1α/HIF2α-ko cells in 1% O_2_ for 48 h and added TMZ (400 μM) to the culture medium for an additional 72 h before detecting apoptosis, and the results showed that there was more late and total apoptosis after HIF1α or HIF2α knockout alone. However, after HIF1α and HIF2α were knocked out simultaneously, the apoptosis rates for early, late and total apoptosis were significantly higher than those of the other groups (Fig 3D). Then, we implanted vector, HIF1α-ko, HIF2α-ko or HIF1α/HIF2α-ko cells (8×10^4^) into mouse brains and treated them with TMZ (2 mg/kg). The results showed that tumor volume and weight were decreased with HIF1α or HIF2α knockout, and the group with simultaneous HIF1α and HIF2α knockout presented the smallest tumor volume and weight (Fig 3E-F). In addition, survival time analysis showed that with the same TMZ (2 mg/kg) treatment in control and HIF1α-ko, HIF2α-ko groups, the survival time became longer after HIF1α-ko or HIF2α-ko, and the longest survival time was observed after the knockout both HIF1α and HIF2α simultaneously (Fig 3G).

### Sox2 was highly expressed in glioma cells

**Figure 4:**
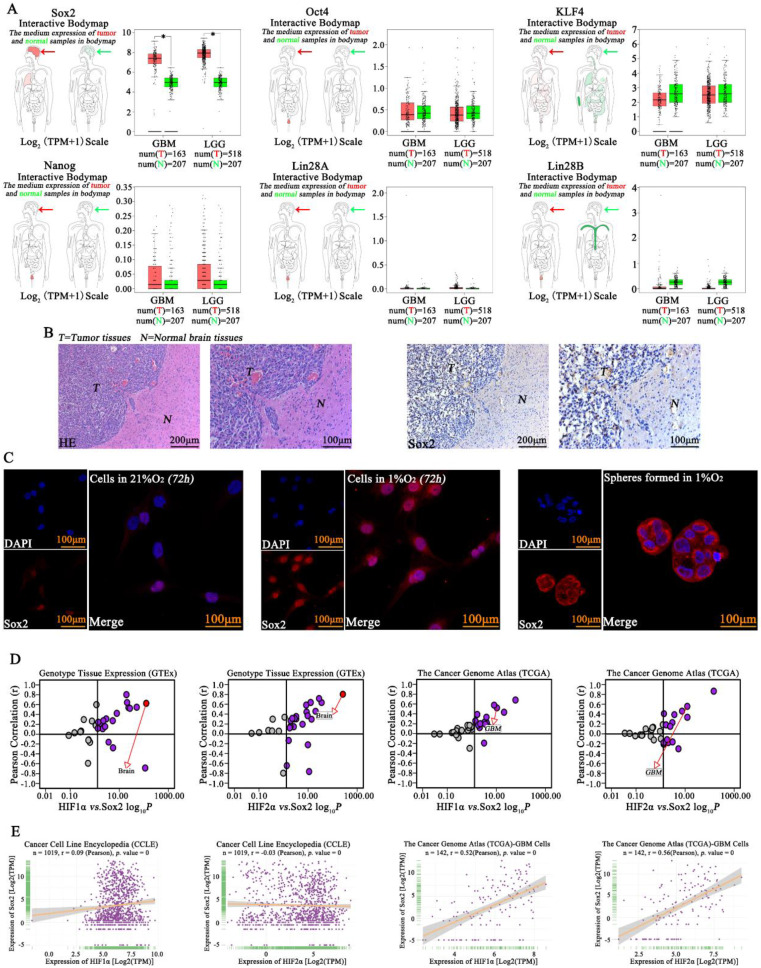
** Sox2 was highly expressed in glioma A** The TCGA database showed that Sox2 expression was high in glioma, higher than that in normal tissues, but hardly any expression of Oct4, Nanog, Lin28A and Lin28B was observed in glioma tissues. **B** Immunohistochemistry revealed high expression of Sox2 in tumors. **C** Immunofluorescence was used for detection and found that cells in a normoxic environment presented a lower expression of Sox2, but Sox2 expression was significantly increased after culturing in 1% O_2_ for 72 h, and spheres formed in 1% O_2_ by single glioma cells also highly expressed Sox2. **D-E** The TCGA, GTEx and CCLE databases revealed that both HIF1α and HIF2α had a positive correlation with Sox2 (^*^P<0.05, independent-samples t test).

Previous studies have revealed the important roles of Sox2, Oct4, KLF4, Nanog, Lin28A and Lin28B in cell pluripotency^20^, and Sox2, Oct4, KLF4, Nanog, Lin28A and Lin28B expression was initially analyzed according to TCGA and GTEx data. The results showed high expression of Sox2 in GBM but hardly showed any expression of Oct4, Nanog, Lin28A or Lin28B, so we evaluated Sox2 in this study. We found that there was higher expression of Sox2 in tumor tissue than in normal tissues (Fig 4A). Next, IHC was used, and high expression of Sox2 was found in tumors; however, there was lower expression of Sox2 in the normal tissues in samples obtained from mouse brains (Fig 4B). Then, immunofluorescence was used for detection, and we found that cells in a normoxic environment presented a lower expression of Sox2, but Sox2 expression increased significantly after cells were cultured in 1% O_2_ for 72 h, and the spheres formed in 1% O_2_ by single glioma cells also highly expressed Sox2 (Fig 4C). Next, according to the TCGA, GTEx and CCLE databases, both HIF1α and HIF2α had a positive correlation with Sox2 (Fig 4D-E).

### Sox2 regulated glioma cell dedifferentiation under hypoxic conditions

**Figure 5:**
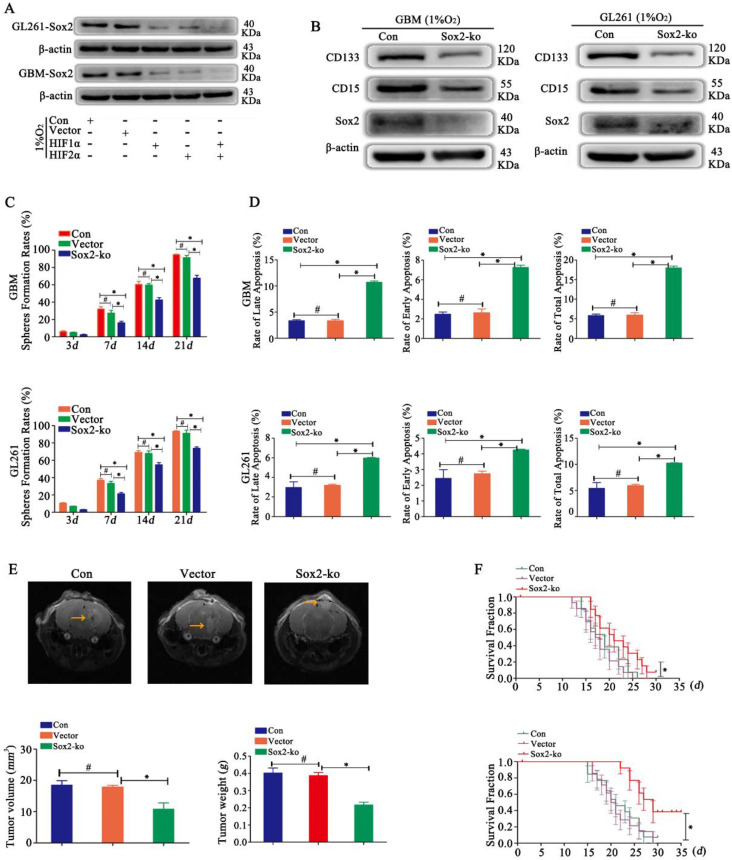
** Sox2 regulated glioma cell dedifferentiation under hypoxic conditions A** Sox2 was highly expressed under hypoxic conditions. After HIF1α or HIF2α knockout, cells showed decreased expression of Sox2, and after knocking out both HIF1α and HIF2α simultaneously, cells presented the lowest expression of Sox2. **B** CD133 and CD15 expression was decreased after Sox2 knockout in CD133^-^CD15^-^ primary and GL261 glioma cells. **C** Sphere formation by single glioma cells was lower after Sox2 knockout than after control transfection. **D** Cell apoptosis detection showed that the apoptosis rate was increased significantly after Sox2 knockout in CD133^-^CD15^-^ primary and GL261 glioma cells. **E-F** The cells described above were implanted into the mouse brain, tumor volume and weight decreased significantly after Sox2 knockout under the same TMZ treatment, and the survival time was prolonged (^*^P<0.05, ^#^*P*>0.05, one-way ANOVA or the log-rank test).

In a hypoxic environment, Sox2 was highly expressed. After HIF1α or HIF2α knockout, cells showed reduced expression of Sox2, and after knocking out both HIF1α and HIF2α simultaneously, cells presented the lowest expression of Sox2 (Fig 5A). Then, we detected CD133 and CD15 expression after Sox2 knockout, and the results revealed that the expression of CD133 and CD15 was reduced (Fig 5B). Then, sphere formation by single glioma cells was detected, and we found decreased sphere formation after Sox2 knockout (Fig 5C). In addition, the results showed that the apoptosis rates for early, late and total apoptosis increased significantly after Sox2 knockout (Fig 5D). Finally, we implanted the cells described above into mouse brains and found that tumor volume and weight were significantly decreased for Sox2-ko cells treated with the same TMZ dose (2 mg/kg), and survival time was prolonged significantly (Fig 5E-F).

## Discussion

There are two kinds of cells in glioma, namely, GSCs and differentiated tumor cells 3, 21. A large number of articles suggest that only GSCs contribute to malignant glioma progression, which leads to a poor prognosis 22, 23. In addition, the hypoxic environment has been suggested to cause glioma to become more malignant through GSC stemness maintenance^24^. For example, in 2009, Li *et al* 25 demonstrated that hypoxia promoted GSC self-renewal and inhibited apoptosis, and this result was confirmed by our previous studies^26^. However, in recent years, an increasing number of studies have found that in addition to GSCs, differentiated glioma cells also play an important role in malignant tumor progression^6^, ^26^, ^27^. Concerning the relationship between GSCs and differentiated glioma cells, previous studies have suggested that a transformation between GSCs and differentiated glioma cells occurs in particular situations, such as hypoxia. For example, Jögi A *et al* found that hypoxia caused increased expression of stem cell markers and promoted tumor formation both in vitro and in vivo^28^; these effects were also demonstrated by Li *et al* in vitro studies that showed hypoxia-induced "dedifferentiation" of differentiated glioma cells, which then acquired stemness features^29^. More recent studies have shown that CD133^-^ differentiated glioma cells derived from GBM-derived neurospheres can be induced to become CD133^+^ GSCs in a hypoxic environment^4^. However, all the cells in these articles were unsorted cells, so we cannot rule out stem cell proliferation. In addition, the culture medium in these studies included EGF and FGF2; thus, we cannot exclude the possibility that the increased rates of GSCs after hypoxia exposure may have been induced by EGF and FGF2, not the hypoxic environment, as EGF and FGF2 can promote the proliferation of stem cells^30^, ^31^. Therefore, to make our results more accurate, we combined CD133^1^, ^32^, ^33^ and CD15^34^, ^35^ as stem cell markers due to the controversy regarding the use of CD133 as a stem cell marker^36^. In addition, we sorted CD133^-^CD15^-^ glioma cells three times and cultured cells under hypoxic conditions without EGF and FGF2. According to our results, GSCs with high expression of CD133 and CD15 could be induced by dedifferentiation of differentiated CD133^-^CD15^-^ cells in the hypoxic environment, and a relatively low apoptosis rate was observed after hypoxia exposure. In addition, the most significant result was found for sphere formation by single glioma cells in 96-well plates. Each well contained only one cell, and almost every survived cell in the hypoxic environment formed spheres with high expression of CD133 and CD15. Undoubtedly, some cells in the wells were GSCs before hypoxia exposure, but the GSC rate was extremely low, and we sorted CD133^-^CD15^-^ cells three times through MACS to minimize this rate. Therefore, dedifferentiation indeed occurs in glioma in hypoxic environments. Previous studies have suggested that the response to hypoxia is mainly mediated by HIF1α and HIF2α, which have been found to have great effects on stemness^37^-^40^. For example, a stem cell marker was found to colocalize with HIF2α, and after HIF2α knockout, self-renewal and proliferation were blocked in vitro, and tumor volume was decreased in vivo. For HIF1α, stemness was also decreased after HIF1α knockout^24^, ^41^, and previous results also indicated that HIF1α regulated the dedifferentiation process under hypoxic conditions^42^; however, few studies have examined whether HIF2α regulates this dedifferentiation process. Therefore, we performed a further study and found that HIF2α was highly expressed under hypoxic conditions, and after HIF2α knockout, the expression of CD133 and CD15 decreased significantly and cell apoptosis was promoted in vitro. Therefore, according to our results, we suggest that GSCs can be induced through dedifferentiation under hypoxic conditions and that both HIF1α and HIF2α regulate this phenomenon.

Next, how HIF1α and HIF2α induce stemness under hypoxic conditions was analyzed. Sox2, Oct4, KLF4, Nanog, Lin28A and Lin28B promote the formation of iPSCs from human dermal fibroblasts^12^, ^13^, especially under hypoxic conditions^14^, which means that Sox2, Oct4, KLF4, Nanog, Lin28A and Lin28B play roles in inducing stemness. In addition, cancer stem-like cells can be induced after reprogramming Sox2 and Oct4 in tumors, such as pancreatic cancer^18^ and melanoma^17^. The same results were demonstrated by Yin *et al*^43^ in 2015, as their results showed that ectopic coexpression of Oct4 and Nanog facilitated hepatocellular carcinoma cell dedifferentiation and the acquisition of features of cancer stem cells, including self-renewal, extensive uncontrolled proliferation, drug resistance and high tumorigenicity. They also found that programming Oct4 and Nanog promoted epithelial-mesenchymal transition changes that contributed to tumor dedifferentiation. Another biological mediator, nitric oxide (NO), has also been suggested to maintain the function of Oct4, thus driving dedifferentiation and inducing the formation of lung cancer stem-like cells^44^. LIN28B is another transcription factor highly expressed in colon cancer; researchers found that this protein had great effects on the dedifferentiation of colon cancer cells. In this research, it was found that colon cancer cells with constitutive Lin28B expression exhibited relatively high expression of the colonic stem cell markers LGR5 and PROM1 and showed an increased probability of tumor recurrence and a reduced patient survival time^45^. Therefore, according to the literature above, we wondered whether Sox2, Oct4, KLF4, Nanog, Lin28A and Lin28B contribute to glioma cell dedifferentiation in hypoxic environments. However, the results from the TCGA databases showed that Sox2 was highly expressed in glioma tissues and that the expression pattern exhibited a large difference between tumor and normal tissues, so we focused on detecting the influence of Sox2 on the dedifferentiation process. Therefore, we knocked out Sox2 and found that CD133 and CD15 expression decreased significantly and that sphere formation by single glioma cells was decreased. In addition, through an in vivo study, we found that tumor formation was impeded. That is, Sox2 regulated glioma cell dedifferentiation under hypoxic conditions. Previous studies have verified that HIF1α acts as an upstream regulator of Sox2 expression^46^; however, there are few reports analyzing whether HIF2α regulates Sox2 and what the effects on dedifferentiation are. Therefore, we researched these issues and found that after HIF1α and/or HIF2α were knocked out, Sox2 expression decreased significantly, especially after knocking out HIF1α and HIF2α simultaneously. According to the results described above, we conclude that HIF1α and HIF2α regulate glioma cell dedifferentiation under hypoxic conditions through Sox2.

**Figure 6:**
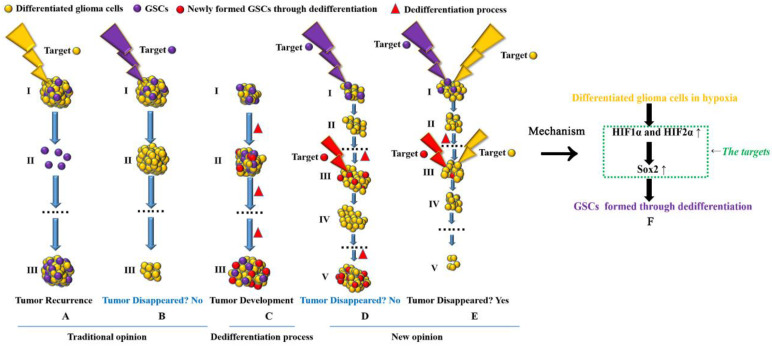
** Targeting both GSCs and differentiated glioma cells to treat glioma A-B** Previous studies suggest that if we target GSCs successfully, tumors will disappear; however, if we target only differentiated cancer cells, tumors are predicted to relapse, as GSCs are the source of cancer recurrence and development. **C** GSCs can be induced to undergo dedifferentiation under hypoxic conditions. **D** If we target only GSCs, tumors cannot disappear. Although we can kill all GSCs in a tumor, new GSCs can be generated through the dedifferentiation of differentiated glioma cells, thus promoting tumor recurrence. **E** Targeting both GSCs and differentiated cancer cells is necessary to eliminate tumors. **F** HIF1α and HIF2α regulate glioma dedifferentiation under hypoxic conditions through Sox2; therefore, HIF1α, HIF2α and Sox2 may be ideal targets for glioma treatment.

Finally, we have to consider new glioma treatment strategies (Fig 6). Previous studies suggest that if we target and kill GSCs completely, tumors can be cured^47^, but there have been no advances in recent years. According to the dedifferentiation process, GSCs can form new tumors and remain malignant; differentiated glioma cells can also form new GSCs through dedifferentiation and promote tumor development. According to the theory above, if we target only GSCs in primary tumors, many differentiated glioma cells remain, and the remaining differentiated cells can form new GSCs through dedifferentiation under hypoxic conditions. Although we can target newly formed GSCs, the number of differentiated tumor cells is much greater than that of GSCs in tumors. Therefore, GSCs can be formed again through continuous dedifferentiation, and the newly formed GSCs will promote new tumor formation. Thus, we should target both GSCs and differentiated tumor cells at the same time, and as a result, tumors can be eliminated. In addition, due to the regulatory mechanism involving the HIF1α/HIF2α-Sox2 pathway in dedifferentiation, all three genes may be useful targets in treating glioma patients.

Briefly, this study demonstrates that both HIF1α and HIF2α, as genes upstream of Sox2, regulate the malignant progression of glioma through dedifferentiation. Therefore, we unexpectedly identified both HIF1α and HIF2α as critical targets in glioma. In addition, Sox2, another factor, was studied in this article and found to be an ideal target for glioma treatment because it was highly expressed in glioma but expressed at low levels in normal tissues. Based on our results, we conclude that the dedifferentiation process is induced under hypoxic conditions via regulation by the HIF1α/HIF2α-Sox2 pathway, which provides new ideal targets for glioma treatment.

## Supplementary Material

Supplementary figure and tables.

## Abbreviations

GSCs: glioma stem cells
HIF1α: hypoxia-inducible factor-1α
HIF2α: hypoxia-inducible factor-2α
TCGA: The Cancer Genome Atlas
GTEx: Genotype-Tissue Expression
CCLE: Cancer Cell Line Encyclopedia
CGGA: Chinese Glioma Genome Atlas
IHC: immunohistochemistry
iPSCs: induced pluripotent stem cells
GBM: glioblastoma multiforme
TMZ: temozolomide
MACS: magnetic activated cell sorting
FBS: fetal bovine serum
GEPIA: Gene Expression Profiling Interactive Analysis
FCM: flow cytometry
PI: propidium iodide
SD: standard deviation
One-way ANOVA: one-way analysis of variance
