# Supplementary material for: HIF1α/HIF2α induces glioma cell dedifferentiation into cancer stem cells through Sox2 under hypoxic conditions
Source: J Cancer. 2022 Jan 1;13(1):1–14. doi: 10.7150/jca.54402 (PMC8692689; doi:10.7150/jca.54402)
Supplement: Supplementary file 1 — Supplementary figure and tables. [file jcav13p0001s1.pdf]

Table S1 the primer sequences of stem cell markers

	upstream (5'→3')	downstream (5'→3')
Human CD133	GCCCCCAGGAAATTTGAGGAAC	GCTTTGGTATAGAGTGCTCAGTGATTG
Human CD15	TGGGCAGGCTGGTCTTGAACT	CACGGCGGCTCACACCTGTA
Mouse CD133	CCGCGATGGACTCTGCTGTTAATG	GGGCACAGTCTCAACATCGTCGTATAC
Mouse CD15	ATCGGGCTGCTGCACACTG	AGCGGAAGTAGCGGCGATAGAC
β-actin	ACCCGCCGCCAGCTCACC	GGGGGGCACGAAGGCTCATC

Table S2 The sequences of sgRNA for knockdown of HIF1α, HIF2α and Sox2

Target	Oligonucleotide sequence(5'-3')
HIF1A	GAACTCACATTATGTGGAAG
HIF2A	CTTGGAGGGTTTCATTGCCG
SOX2	AAAGTTTCCACTCGGCGCCC

abbreviation	Full Name
O	oligodendrocytoma
A	astrocytoma
rO	Recurrent oligoastrocytoma
rA	Recurrent astrocytoma
AO	Anaplastic oligodendrocytoma
AA	Anaplastic astrocytoma

Table S3 The abbreviations and full names for materials used in histological experiments in the article

rAA	Recurrent anaplastic astrocytoma
GBM	Glioblastoma multiforme
rGBM	Recurrent glioblastoma multiforme

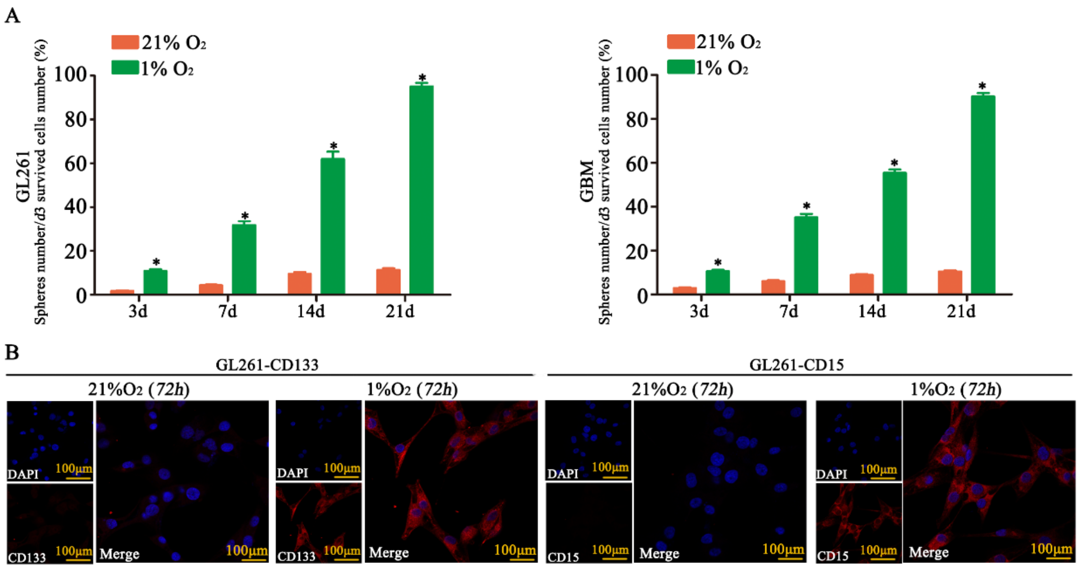

Supplementary Figure 1 **A** Just one $CD133^-CD15^-$ GL261 or primary glioma cell was plated in each well of 96-well plates and incubated in 21% O_2 or 1% O_2. Most of the cells died in 21% O_2; however, the cells in 1% O_2 appeared to be in a state of suspended spheres after hypoxic exposure for 7 days and the sphere formation rate reached to 60% under hypoxic conditions for 14 days and its rate was over 95% after hypoxic exposure for 21 days. **B** $CD133^-CD15^-$ GL261 cells presented high expression of CD133 and CD15 after culturing in 1% O_2 for 72 h, but there was no expression in the cells cultured in 21% O_2 for 72h.
